# The role of allied ophthalmic personnel in achieving universal eye health coverage in South Asia

**Published:** 2020-12-31

**Authors:** YD Sapkota

**Affiliations:** 1 Regional Programme Manager, South East Asia: International Agency for Prevention of Blindness, Nepal.

**Systematic and dedicated training; setting up of regulatory bodies to monitor training, and accreditation is the need of the hour in improving eye care resources in South Asian nations.**

**Figure:**
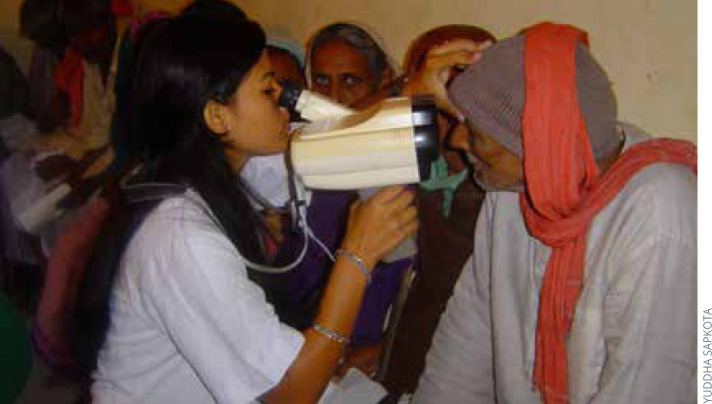
An ophthalmic assistant performs auto refraction, in Sagarmatha Chaudhary Eye Hospital. **LAHAN, NEPAL**

The need for expansion of eye care services, primary eye care, early detection of blinding and potentially blinding conditions and injury is well-recognised. However, achieving these goals is not easy, given the limited availability of ophthalmologists. To tackle diseases and disorders that affect people's vision, and to implement comprehensive eye care, sufficient availability of the eye care personnel at all levels is necessary.

The concept of dedicated training Allied Ophthalmic Personnel (AOP) in South Asia, started in India and Nepal in 1980.^1^ The training and development of eye care resources at this level went on to become the backbone of primary, and to some extent, secondary eye care services delivery systems in South Asian nations.

The Allied Ophthalmic Personnel (AOP) deliver primary eye care and also help increase the productivity of the ophthalmologists. The training programme for the eye care personnel expanded to other South Asian countries, covering ophthalmic assistants, ophthalmic nurses, mid-level ophthalmic personnel, ophthalmic technologists, and vision technicians. These professionals have contributed to the expansion and growth of eye care services, besides playing a key role in the development of both specialised and primary eye care services in all the countries of the region.

In Nepal and Bhutan, a school graduate with ten years of schooling^2^ and three years' training in ophthalmology, will perform the following tasks:

- Diagnose and initiate treatment/appropriate management for most common eye problems including refractive errors
- Recognise conditions that require a higher level of care and refer such cases to an ophthalmologist
- Organise and run outreach activities such as screening camps and school health programmes
- Impart eye health education, health promotion and prevention of preventable eye diseases
- Select and prepare patients who require eye surgery
- Assist the ophthalmologist during surgery
- Provide postoperative care
- Perform practical procedures involved in the examination and treatment of common eye problems
- Carry out visual acuity assessment refraction and prescription of spectacles
- Perform simple assessment of low vision and suggest necessary interventions, devices, and aids
- Counsel patients and market services
- Manage the eye clinic, including record keeping and supplies
- Supervise and train community-level health care personnel in primary eye care.

In Bangladesh, India, and Sri Lanka, most of these tasks are included in the training given to the AOPs. However, these AOPs are not allowed to work independently and prescribe drugs.

In Sri Lanka and Bangladesh, the training programmes for AOPs and optometrists are not recognised by government. Also, there is no accreditation nor any regulatory body for the purpose. In India, AOPs are trained on the basis of need. There is no uniform training curriculum or monitoring by any regulatory body. However, the country is in the process of considering these aspects under the proposed ‘Allied and Healthcare Professions Bill, 2018’.

In the Maldives, where ophthalmologists and optometrists generally render the eye care services, there are no ophthalmic assistants or AOPs.

The Nepal Health Professional Council, set up by the government of Nepal, has issued the code of ethics and job description, to regulate the training programme for AOPs and their tasks across the country.

**Figure 2:**
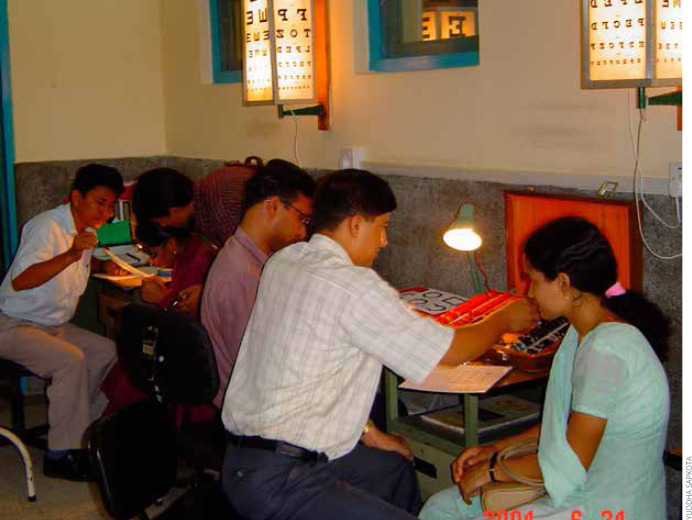
An ophthalmic assistant performs refraction, in Nepal Eye Hospital, **KATHMANDU NEPAL**

In Bhutan, the government recognises the importance of AOPs and is in the process of forming a regulatory body under the ministry of health.

With the effective utilisation of AOPs in Nepal, the productivity of ophthalmologists has gone up. Approximately 200 active ophthalmologists perform more than 300,000 cataract operations every year. The expansion of vision assessment and refraction services to all 77 districts of the country can be credited to AOPs.

Although AOPs have proven to play an important role in efficient eye care service delivery in most South Asian countries, some challenges still need to be addressed:

- the absence of a proper body within the governments to ensure the quality of training, monitoring, and accreditation system for ongoing training programmes
- absence of a uniform code of ethics, job descriptions and training curriculum
- lack of defined career pathways for AOPs
- absence of a professional body for human resources, at this level.

AOPs have always demonstrated their importance in eye care and can play a significant part in achieving the universal eye health coverage, as suggested by WHO. AOPs must be formally recognised, and their functions regulated in all the countries of South Asia.
